# Temporal and Functional Profiling of the Microbiome of High and Low Nitrogen Content Barley Seed in Silo Storage

**DOI:** 10.1002/fsn3.72179

**Published:** 2026-08-10

**Authors:** Kalonji A. Tshisekedi, Tim Van Den Bossche, Lennart Martens, Pieter De Maayer, Angela Botes

**Affiliations:** ^1^ School of Molecular and Cell Biology University of the Witwatersrand Johannesburg South Africa; ^2^ Sydney Brenner Institute for Molecular Bioscience, Faculty of Health Sciences University of the Witwatersrand Johannesburg South Africa; ^3^ VIB‐UGent Center for Medical Biotechnology, VIB Ghent Belgium; ^4^ Department of Biomolecular Medicine, Faculty of Medicine and Health Sciences Ghent University Ghent Belgium; ^5^ BioOrganic Mass Spectrometry Laboratory (LSMBO), IPHC UMR 7178, University of Strasbourg, CNRS Strasbourg France; ^6^ Infrastructure Nationale de Protéomique ProFI─FR2048 Strasbourg France

## Abstract

Barley grain quality is influenced by nitrogen content and storage conditions; however, their impact on the composition and function of the grain microbiome is not well understood. This study combined metataxonomic (16S rRNA and ITS) profiling, metagenome sequencing, and metaproteome analyses to characterize the structure and function of the barley grain microbiome. Grains with high (> 1.5%) and low (< 1.5%) nitrogen content from a single barley cultivar (Kadie) were sampled at harvest and after 3, 6, and 9 months of storage. Amplicon sequencing revealed a community dominated by Proteobacteria, Firmicutes, and Ascomycota, while metagenomics confirmed the abundance of genera such as *Erwinia*, *Pantoea*, and *Pseudomonas*, aligning with previous reports of barley endophytes. While a consistent set of core microbial genera was identified, their relative abundances varied throughout storage. Metagenomic analysis revealed the high‐nitrogen grain microbiome had potential for rapid metabolic activity that declined post‐harvest, whereas the low nitrogen grain community sustained prolonged metabolic potential. Metaproteomics confirmed that these functional shifts revealed a temporal transition from active growth to stress tolerance. Findings from this work contribute to a better understanding of the barley grain microbiome during prolonged storage, offering insights that could help optimize storage for malting and brewing.

## Graphical abstract

Combined metataxonomic, metagenomic, and metaproteomic profiling of barley grain across 9 months of storage reveals a core microbiome characterized by Proteobacteria, Firmicutes, and Ascomycota, with genera such as *Erwinia*, *Pantoea*, and *Pseudomonas* persisting throughout. Nitrogen content shapes microbial functional trajectory: high nitrogen content grain supports rapid metabolic activity that declines after harvest, while low nitrogen content grain sustains prolonged metabolic potential. Metaproteomics confirms this as a temporal transition from active growth to stress tolerance. These findings offer a functional roadmap for optimizing barley storage conditions for malting and brewing.

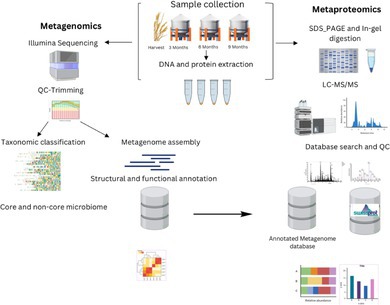

## 1 Background

Barley ( *Hordeum vulgare* L.) is a widely cultivated cereal crop valued for its adaptability to diverse growing conditions (Asare et al. 2011; Hornsey 2003). Its unique enzymatic and physical characteristics make it the preferred grain for beer production (Barnes 2017; Briggs et al. 1981). The quality of barley grain plays a key role in determining the flavor profile of both malt and beer (Bamforth 2017). Compromised barley quality, due to suboptimal cultivation and storage, directly affects the brewing process, producing beer with poor flavor properties and concomitant economic losses (Bamforth 2017; Briggs et al. 2004).

Following harvest, barley seeds may be stored for a period of time before malting to break seed dormancy (Gupta et al. 2010; Felšöciová et al. 2021). Proper storage conditions are essential for preserving the physical and biochemical properties of barley required for brewing and to control microbial growth, which may impact beer quality (Bokulich and Bamforth 2013). These include maintaining low humidity, low temperatures, and adequate ventilation (Felšöciová et al. 2021; Abubakar 2019). High humidity can encourage fungal growth, while elevated temperatures further exacerbate microbial activity (Pitt and Hocking 1997; Suzuki 2020). Condensation inside silos often creates “hot spots,” providing ideal conditions for microorganisms to thrive. These conditions lead to spoilage, causing changes in the color, odor, and flavor of the grain (Bingham et al. 2012; Felšöciová et al. 2021).

The nitrogen content of barley grain is also a critical factor in the quality of beer (Adeboye et al. 2011). Grain with a lower nitrogen content is preferable, as a higher nitrogen content is indicative of higher protein levels (Agu and Palmer 2001), which can result in undesirable foam stability and off‐flavors in the beer (Ahmed et al. 2018). Microorganisms play an important part in the nitrogen cycle, turning atmospheric nitrogen into usable forms and recycling it through ecosystems, thus impacting the nitrogen content of crops such as barley (Agu and Palmer 2001). Their activities ensure the availability of appropriate nitrogen sources, which is essential for optimizing barley grain quality for beer production (Baslam et al. 2021). While the impact of nitrogen content on beer quality is well‐documented, the relationship between the barley nitrogen content and microbial dynamics during storage is less understood.

The barley microbiome comprises a diverse community of bacteria, fungi and viruses that influences the grain properties (Bziuk et al. 2021; Laitila et al. 2018; Li et al. 2018). The presence of fungi is especially problematic as they not only cause spoilage but may also introduce mycotoxins, which pose a health risk to both humans and animals (Bingham et al. 2012; Felšöciová et al. 2021). Bacterial communities associated with barley seeds have been relatively understudied. A recent study has shed light on the bacterial endophytes present in barley seeds, revealing a core microbiome dominated by genera such as *Curtobacterium*, *Paenibacillus* and *Pantoea* (Bziuk et al. 2021). These bacterial endophytes have been shown to promote plant growth and enhance resistance against phytopathogens (Bziuk et al. 2021; Li et al. 2018).

Previous research on cereals such as maize and wheat has shown that storage conditions influence microbial diversity; for instance, prolonged maize storage reduces microbial richness and increases grain susceptibility to fungal colonization (Brito et al. 2022; Lane et al. 2018). Similarly, the microbiome composition affects dormancy and germination in wheat, while studies on rice report declining beneficial microorganisms during storage, raising spoilage risks (Qiu et al. 2024; Kuźniar et al. 2020). By contrast, little is known about how storage impacts the barley seed microbiome, limiting the optimisation of post‐harvest handling and grain quality assurance.

This study investigates the evolution of the barley grain microbiome during long‐term storage (up to 9 months) in grain with high and low nitrogen content, by integrating taxonomic, metagenomic, and metaproteomic analyses. While earlier research has mainly focused on single time‐point assessments, this approach provides a comprehensive understanding of microbial succession and functional shifts across both nitrogen levels throughout prolonged silo storage.

## 2 Methodology

### 2.1 Sample Collection

A single variant (Kadie) of dry barley grain was sampled from select Anheuser‐Busch InBev (AB‐InBev) storage facilities in the Western Cape Province, South Africa (Table 1). Sampling was carried out at four time points: at harvest, and after 3, 6 and 9 months of storage. At each time point, grains were classified by AB‐InBev as either high grain nitrogen content (> 1.5%) or low grain nitrogen content (< 1.5%), with total nitrogen quantified in‐house using the Dumas combustion method (Krausová et al. 2018; Kupetz et al. 2018). A total of three replicate samples were collected for both high and low nitrogen content grains at each of the four time points (at harvest, and after 3, 6 and 9 months of storage), resulting in a final batch of 24 samples (Table 1). The samples were collected in sterile bags and stored at −18°C until microbial DNA extraction.

**TABLE 1:** PERMANOVA testing the effects of storage duration and grain nitrogen content on barley grain microbiome composition.

Factor	Bacteria (16S rRNA)				Fungi (ITS1)			
	df	*F*	*R* ^2^ (%)	*p*	df	*F*	*R* ^2^ (%)	*p*
Storage duration	**3**	**1.600**	**21.030**	**0.067**	**3**	**7.950**	**55.660**	**0.001**
Grain nitrogen content	1	0.250	1.240	0.989	1	2.680	11.340	0.054
Storage duration × grain nitrogen content	**3**	**2.410**	**26.480**	**0.003**	**3**	**2.160**	**10.300**	**0.055**
Residual	14		51.390		15		23.860	

*Note:* PERMANOVA results for the effect of storage duration and grain nitrogen content on barley grain microbiome composition. Bray–Curtis dissimilarity, 999 permutations (adonis2). Bacterial community: 16S rRNA amplicon sequencing; fungal community: ITS1 amplicon sequencing. df, degrees of freedom; *F*, pseudo‐*F* statistic; *R* ^2^, proportion of variance explained (%); *p*, permutation *p*‐value. Bold: *p* < 0.05.

### 2.2 DNA Extraction, Amplification

Approximately 10 g of each barley sample was crushed using a sterile mortar and pestle. The crushed material was suspended in 40 mL of phosphate‐buffered saline (PBS; pH 7.4, 0.137 M NaCl, 0.01 M Na_2_HPO_4_, and 0.0018 M KH_2_PO_4_). The suspensions were sonicated for 7 min at 18 W amplitude, using a 30‐s on–off pulse cycle with a Microson Ultrasonic Cell Disruptor (Wazobia Enterprise, USA). After sonication, the mixtures were centrifuged at 4000 × *g* for 1 min to separate the supernatant from the solid residue. The supernatants were passed through a 0.45 μm pore Sartorius‐Stedim Biotech filter membrane. Microbial cells retained on the filter membrane were transferred to sterile Eppendorf tubes. Metagenomic DNA (mDNA) was extracted from these samples using the ZymoBIOMICS DNA/RNA Miniprep Kit (Zymo Research, USA) following the manufacturer's instructions.

The samples were submitted to Molecular Research LP (www.mrdnalab.com) for amplicon and metagenomic sequencing. The V1–V3 regions of the 16S rRNA gene were amplified using the universal primers 27F (5′‐AGRGTTTGATCMTGGCTCAG‐3′) and 519R (5′‐GTNTTACNGCGGCKGCTG‐3′) (Valverde et al. 2016). Fungal internal transcribed spacer region 1 (ITS1) was amplified using the primers ITS1 (CTTGGTCATTTAGAGGAAGTAA)/ITS4 (GCTGCGTTCTTCATCGATGC) (Kuang et al. 2021).

### 2.3 Metagenomic Sequencing and Quality Check

For metagenome sequencing, paired‐end sequencing (2 × 250 bp) was conducted on an Illumina NovaSeq 6000 platform. The raw sequencing reads underwent initial quality assessment using FastQC (v0.12.1) (Andrews 2010) and MultiQC (v1.15) (Ewels et al. 2016). Low‐quality sequences were filtered using Trimmomatic (Bolger et al. 2014), with reads shorter than 36 bp or those exhibiting an average Phred quality score below 20 being removed. To identify host‐derived DNA contamination, retained reads were aligned against the barley reference genome ( *H. vulgare* , assembly GCF_904849725.1) using Bowtie2 (v2.5.1) (Langmead and Salzberg 2012). Alignment outputs were processed with SAMtools (v1.19) (Li et al. 2009), with unaligned reads retained for subsequent microbial analyses. Reads successfully mapped to the barley genome were categorized as host contamination and excluded from further downstream processing.

### 2.4 Metataxonomic Analysis

Amplicon sequences were processed using the DADA2 pipeline (Callahan et al. 2016), which performs quality filtering, trimming, denoising, merging of paired‐end reads, and removal of chimaeric sequences to infer exact amplicon sequence variants (ASVs). Taxonomic assignment was conducted against the SILVA database v138.2 for bacterial 16S rRNA gene sequences and the UNITE database v10.0 for fungal ITS1 sequences (Quast et al. 2013). Sequencing depth across bacterial samples ranged from 2737 to 23,240 reads. Sample BCT1 was identified as a low‐depth outlier (2737 reads), considerably below the distribution of remaining samples (range: 15,241–23,240 reads). A rarefaction threshold of 15,000 reads per sample was selected to retain the maximum number of samples while ensuring adequate coverage for diversity estimation; this threshold resulted in the exclusion of BCT1 only. Re‐extraction and re‐sequencing of BCT1 was not feasible due to insufficient remaining sample material from the brewing facility. All downstream analyses were conducted on the 23‐sample rarefied dataset.

Alpha diversity was quantified using observed ASV richness, the Chao1 richness estimator, and Shannon diversity index. Normality within storage‐time groups was assessed using the Shapiro–Wilk test (González‐Estrada et al. 2022; Shapiro and Polz 2014). As several groups deviated from normality, differences among storage time and nitrogen categories were evaluated using the Kruskal–Wallis test, followed by Dunn's post hoc comparisons with Benjamini–Hochberg correction where appropriate (Kruskal and Wallis 1952). Beta diversity was assessed using Bray–Curtis dissimilarity calculated from rarefied abundance tables (Bray and Curtis 1957). Community composition was tested using permutational multivariate analysis of variance (PERMANOVA; adonis2, vegan v2.6, 999 permutations) (Anderson 2017). Homogeneity of multivariate dispersion was verified using betadisper prior to interpreting PERMANOVA results. Relative abundances were calculated from the complete community dataset prior to visualization; taxa displayed in figures represent the most abundant genera, but percentages reflect proportions relative to total sample reads.

### 2.5 Metagenomic Analysis

The metagenomic reads were profiled using Kraken2 v2.1.3 (Wood et al. 2019) with default parameters. This analysis leveraged the NCBI RefSeq reference database (Release 202) to assign microbial classifications. To enhance the precision of abundance estimates, Bracken v2.7.0 (Lu et al. 2017) was subsequently applied, statistically adjusting read counts at species and genus levels. Contigs were assembled from trimmed reads using MetaSPAdes v3.15.3 (Nurk et al. 2017), with assembly quality evaluated using QUAST v5.2.0 (Gurevich et al. 2013). Gene prediction was performed using Prokka v1.14.5 (Seemann 2014). Functional annotation was conducted using eggNOG‐mapper v 1.0.3 against the eggNOG v5.0 database (Huerta‐Cepas et al. 2019). Annotation results were subsequently organized into Kyoto Encyclopedia of Genes and Genomes (KEGG) orthology (KO) profiles (Ogata et al. 1999), Clusters of Orthologous Groups (COG) functional categories (Tatusov et al. 2003) and Carbohydrate‐Active enZymes (CAZy) classifications (Yin et al. 2012). For comparing functional profiles across different sample groups, the relative abundance of CDS within each COG category was standardized using a *Z*‐score transformation. These *Z*‐scores were calculated in R and subsequently used to generate heatmaps for visualization.

### 2.6 Protein Extraction, Digestion and Liquid Chromatography–Mass Spectrometry

For each sample, ~10 g barley seeds were ground, suspended in 40 mL PBS (pH 7.4) and sonicated at 18 W for 7 min (30 s on–off). After centrifugation (4000 × *g*, 1 min), the supernatant was filtered (0.45 μm pore). Proteins were extracted by resuspending microbial cells (filter retentate) in 50 mM Tris buffer (pH 7.5) containing 1% (w/v) SDS, followed by vortexing (2 min), sonication (5 min on ice, 20% amplitude), boiling (95°C, 15 min) and gentle shaking (4°C, 1 h, 20 rpm). After centrifugation (1000 × *g*, 5 min), proteins in the supernatant were precipitated overnight in cold acetone (5:1 acetone to sample), pelleted (1000 × *g*, 1 min, 4°C), dried on ice and resuspended in 50 mM Tris (pH 7.4). A 30 μL aliquot was subjected to SDS‐PAGE with 12% (w/v) polyacrylamide gels stained with Coomassie Brilliant Blue (G‐250, Bio‐Rad). Gel fragments were submitted to the Council for Scientific and Industrial Research (CSIR) in Pretoria, South Africa, for in‐gel digestion and liquid chromatography–mass spectrometry (LC–MS).

Five gel bands per sample were excised and transferred to individual 1.5 mL microcentrifuge tubes. To each tube, 200 μL of 10 mM DTT in 25 mM NH_4_HCO_3_ was added, and the samples were reduced at 60°C for 1 h. Following reduction, the supernatant was removed, and the gel pieces were alkylated by adding 200 μL of 55 mM iodoacetamide in 25 mM NH_4_HCO_3_ and incubating in the dark for 30 min at room temperature. After alkylation, the solution was discarded, and the gel pieces were washed twice with 250 μL of 25 mM NH_4_HCO_3_ for 15 min each, followed by two washes with 250 μL of acetonitrile for 10 min each. The gel pieces were then dehydrated by adding 200 μL of acetonitrile and incubating for 10 min, after which the acetonitrile was removed, and the gel pieces were air‐dried. Gel pieces were digested overnight at 37°C with trypsin (0.25% v/v). Reactions were halted using 0.1% (v/v) formic acid and dried under vacuum. Peptides were resuspended in 2% acetonitrile, 0.2% formic acid and analyzed on a Dionex Ultimate 3000 RSLC system (ThermoFisher, USA) coupled to an AB Sciex 5600 TripleTOF mass spectrometer (Sciex, USA). Data‐dependent acquisition was performed in positive ion mode with full MS scans (m/z 400–1500) followed by 40 MS/MS scans (m/z 100–1800).

### 2.7 Database Generation, Protein Identification and Annotation

A customized database (372,508 proteins; 745,016 in concatenated target–decoy format) comprising host ( *H. vulgare* ) proteins, in‐house contaminants (CSIR), and metagenome‐annotated proteins was generated. Protein identification was performed using Protein Pilot v5.010 (Seymour and Hunter 2015) by processing raw (.wiff) data against the customized database. A target–decoy approach was employed for false discovery rate (FDR) control (Aggarwal and Yadav 2016). Protein hits were filtered based on the Unused ProtScore, set to achieve a protein‐level FDR of ≤ 1% (Seymour and Hunter 2015). Further stringency was applied, retaining only proteins with a confidence score ≥ 95% and a contribution score ≥ 0.1 (10%). Host and contaminant proteins were subsequently removed to focus on the microbial metaproteome.

Peptides were identified and functionally annotated using the Unipept web application v5 (Vande Moortele et al. 2025), which exclusively processed fully tryptic peptides. For each peptide, taxonomic lineages were assigned using a Lowest Common Ancestor (LCA) analysis, and associated functional annotations, including Enzyme Commission (EC) numbers and Gene Ontology (GO) terms, were retrieved directly from UniProtKB (Vande Moortele et al. 2025; Verschaffelt et al. 2021).

## 3 Results

### 3.1 Bacterial and Fungal α‐Diversity

**FIGURE 1:**
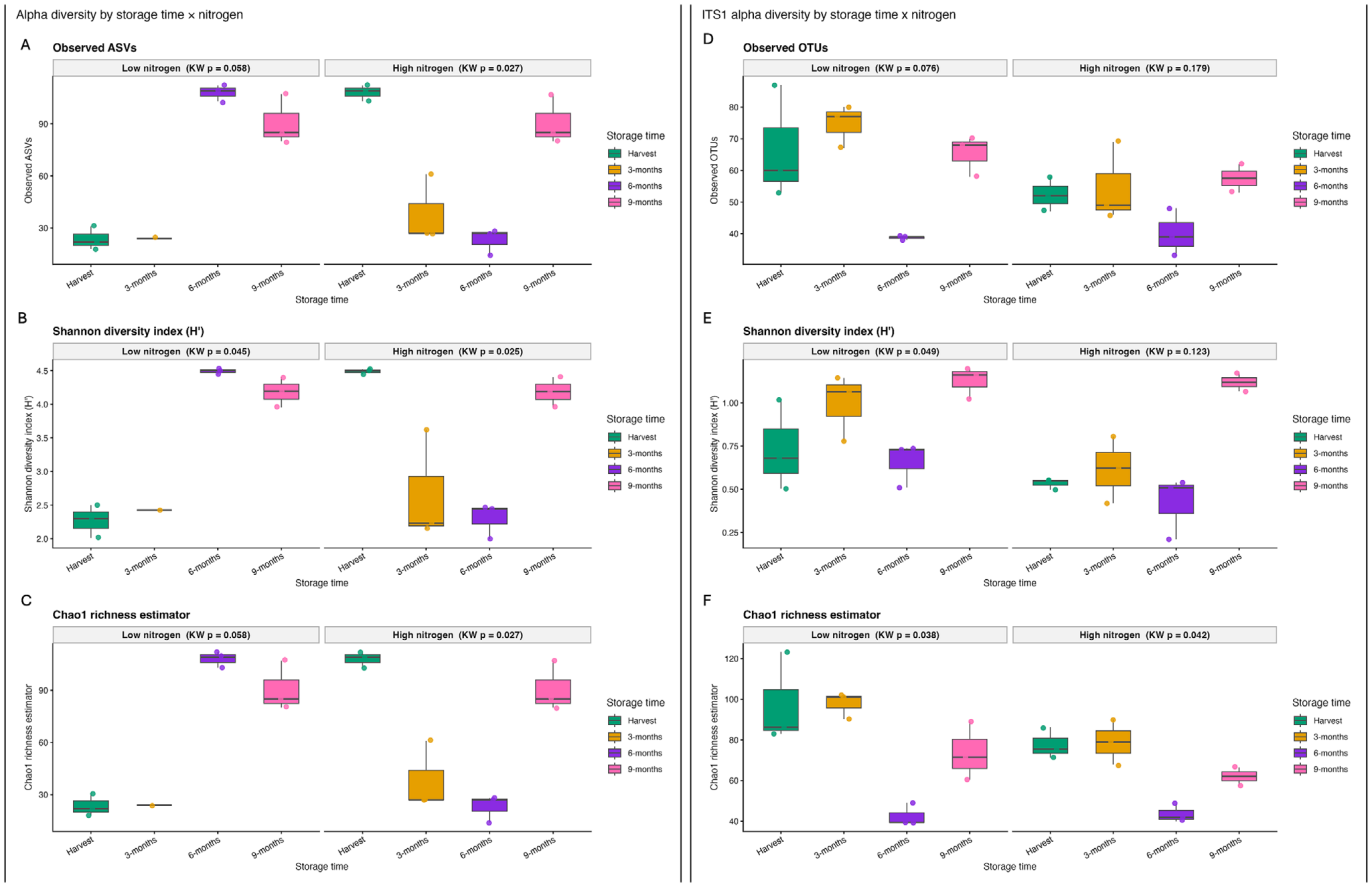
Alpha diversity of the bacterial and fungal communities in stored barley grain across storage time and grain nitrogen content. (A) Observed ASVs, (B) Shannon diversity index (*H′*), and (C) Chao1 richness estimator for the bacterial community (16S rRNA gene amplicon sequencing). (D) Observed OTUs, (E) Shannon diversity index, and (F) Chao1 richness estimator for the fungal community (ITS1 amplicon sequencing).

The amplicon datasets (16S and ITS) were processed using DADA2 and subsequently rarefied to 15,000 and 220,000 reads per sample, respectively. This resulted in a total of 23 samples for each dataset, as one bacterial sample (BCT1) and one fungal sample (IPOT3) were dropped for falling below these thresholds. After filtering, the bacterial dataset comprised 472 ASVs, while the fungal dataset contained 219 ASVs. Bacterial diversity was assessed using observed richness, Chao1 and Shannon indices. Because several groups deviated from normality (Shapiro–Wilk *p* < 0.05; Table S1), non‐parametric tests were used for all bacterial comparisons. Storage duration and grain nitrogen content had no significant individual impact on bacterial richness or diversity (*p* > 0.820; Table S2). When tested within each nitrogen group separately, storage duration significantly influenced bacterial α‐diversity in both low‐nitrogen (Kruskal–Wallis, *p* = 0.040) and high‐nitrogen samples (*p* = 0.027; Table S2). The most pronounced divergence between groups occurred at 6 months of storage, when low‐nitrogen grain diversity had rebounded to harvest‐level values (Observed ASVs: 103–112; Shannon: 4.44–4.53), while high‐nitrogen grain diversity was at its minimum (Observed ASVs: 14–28; Shannon: 1.99–2.47), before the two groups converged again by 9 months (Figure 1A–C).

At harvest, barley grain with a high nitrogen content hosted greater microbial diversity than grain with low nitrogen content (Observed ASVs: 103–112 vs. 18–31; Shannon index: 4.44–4.52 vs. 2.01–2.50; Table S2 and Figure 1A–F). Within the low nitrogen group, α‐diversity remained low through the initial 3 months of silo storage but increased by the 6‐month time point (Observed 103–112; Shannon 4.44–4.53; Table S1 and Figure 1A–F). This richness persisted through 9 months storage, despite a minor contraction in ASV counts (Observed 80–107; Shannon 3.95–4.40; Table S1 and Figure 1A–C). In contrast, high nitrogen grain yielded their highest diversity at harvest, which subsequently dropped after 3 months in the silo (Observed 27–61; Shannon 2.15–3.62; Table S1 and Figure 1A–C) and reached a minimum at the 6‐month interval (Observed 14–28; Shannon 1.99–2.47; Table S1 and Figure 1A–C). At 9‐month storage, diversity in these high nitrogen samples recovered (Observed 80–107; Shannon 3.95–4.40; Table S1 and Figure 1A–C) to match the low nitrogen group. Consequently, both nitrogen categories converged on comparable microbial richness and diversity ranges by the conclusion of the storage period.

Fungal α‐diversity fluctuated significantly across the storage period for observed richness (Kruskal–Wallis, *p* = 0.007), Shannon index (*p* = 0.007) and Chao1 estimates (*p* = 0.001; Table S3). While grain nitrogen content alone did not significantly shift fungal diversity (*p* > 0.096), storage duration exerted a consistent influence when analyzed within both the high and low nitrogen groups (all *p* < 0.050; Table S1). At harvest, grain with low nitrogen content hosted greater fungal richness (Observed 53–87; Table S1 and Figure 1D–F) and diversity (Shannon 0.50–1.14; Table S1 and Figure 1D–F) than high nitrogen grain (Observed 47–58; Shannon 0.50–0.55; Table S1 and Figure 1D–F). Community complexity peaked after 3 months of storage, an effect most pronounced in the low nitrogen group (Observed 67 to 80; Shannon 0.78 to 1.14; Table S1 and Figure 1D–F). However, richness dropped at the 6 months storage in both groups (Observed 38–39 in low nitrogen; 33 to 48 in high nitrogen; Table S1 and Figure 1D–F), representing a significant decline from harvest values (*p*_adj_ = 0.015) and the 3 months peak (*p*_adj_ = 0.007). After 9 months of storage, fungal richness and diversity increased again (*p*_adj_ = 0.005). At this stage, low nitrogen grain maintained slightly higher richness (Observed 58–70; Table S1 and Figure 1D–F) than high nitrogen grain (Observed 53–62; Table S1 and Figure 1D–F), though Shannon diversity reached comparable values between the two groups (1.02–1.20 and 1.07–1.17, respectively; Table S1 and Figure 1D–F).

### 3.2 Bacterial and Fungal β‐Diversity

Differences in bacterial and fungal community structure across storage duration and grain nitrogen content were assessed using PERMANOVA based on Bray–Curtis dissimilarity. Bacterial community structure was significantly affected by the interaction between storage duration and grain nitrogen content, which accounted for the largest proportion of explained variance in the bacterial dataset (*R* ^2^ = 26.48%, *F* = 2.405, *p* = 0.003; Table 1). Storage duration alone accounted for a smaller proportion of the variance (*R* ^2^ = 21.03%, *F* = 1.598, *p* = 0.067), while grain nitrogen content alone had no detectable effect (*R* ^2^ = 1.24%, *F* = 0.250, *p* = 0.989; Table S2). Analysis of similarities (ANOSIM) supported differentiation by storage duration (*R* = 0.179, *p* = 0.040) but not by grain nitrogen content (*R* = −0.074, *p* = 0.925). Multivariate dispersion differed significantly across storage durations (betadisper: *F* = 12.02, *p* < 0.001), with mean distance to centroid declining from 0.568 at harvest to 0.348 at 9 months, but did not differ between grain nitrogen content groups (*F* = 0.286, *p* = 0.599). Pairwise PERMANOVA comparisons indicated that bacterial communities at 9 months differed significantly from all earlier time points: harvest (*p*_adj_ = 0.009), 3 months (*p*_adj_ = 0.014), and 6 months (*p*_adj_ = 0.009; Table S2). No significant differences were observed between any other pair of time points (e.g., harvest versus 3 months, *p*_adj_ = 0.929; Table S2).

In contrast, fungal community structure was primarily affected by storage duration, which accounted for 55.66% of the variance (*F* = 7.951, *p* = 0.001). Grain nitrogen content and its interaction with storage duration explained less variance (*R* ^2^ = 11.34%, *F* = 2.685, *p* = 0.054; and *R* ^2^ = 10.30%, *F* = 2.159, *p* = 0.055, respectively) and were not significant at *p* < 0.05. ANOSIM confirmed separation by storage duration (*R* = 0.514, *p* = 0.001) but not by grain nitrogen content (*R* = 0.082, *p* = 0.084), and no differences in multivariate dispersion were detected for either factor (betadisper: storage *F* = 0.418, *p* = 0.742; nitrogen *F* = 2.531, *p* = 0.127). Pairwise PERMANOVA comparisons showed no significant difference between harvest and 3 months samples (*p*_adj_ = 0.130), whereas 6 months (*p*_adj_= 0.006) and 9 months communities (*p*_adj_ = 0.006) differed significantly from harvest, and 9 months samples also differed from both 3 months (*p*_adj_ = 0.006) and 6 months communities (*p*_adj_ = 0.006). The 3 months versus 6 months comparison was marginal and did not reach significance after correction (*p*_adj_ = 0.056; Table S2).

**FIGURE 2:**
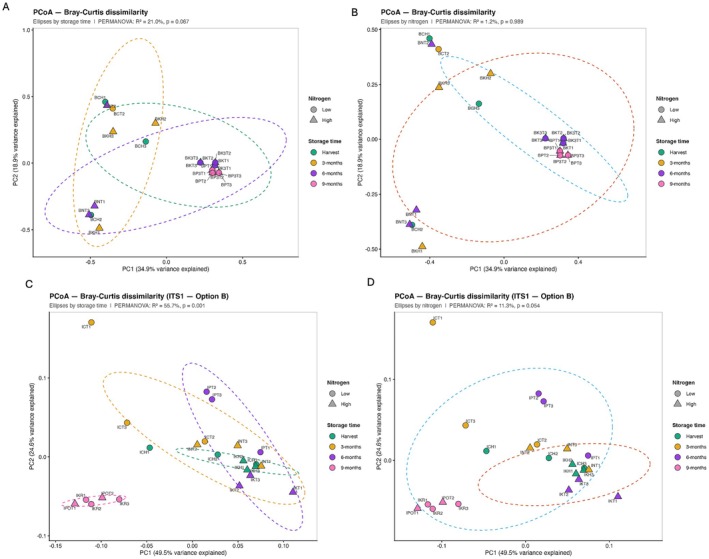
Principal coordinates analysis (PCoA) of Bray–Curtis dissimilarity for bacterial and fungal communities in stored barley grain. (A, B) Bacterial community (16S rRNA gene amplicon sequencing); ellipses grouped by (A) storage time and (B) nitrogen grain content. (C, D) Fungal community (ITS1 amplicon sequencing); ellipses grouped by (C) storage time and (D) nitrogen grain content.

Ordination of Bray–Curtis dissimilarities by PCoA showed distinct temporal patterns in both bacterial and fungal datasets (Figure 2). In the bacterial dataset, the first two axes explained 34.9% and 18.9% of the variance, with samples distributed along a temporal gradient on PC1 (Figure 2A,B). Harvest and 3 months communities were positioned predominantly at negative PC1 values, while 6 and 9 months communities shifted towards positive PC1 values (Figure 2A,B). Samples at 9 months formed a tight cluster irrespective of grain nitrogen content, whereas earlier time points were more dispersed and partially overlapped. In the fungal dataset, the first two axes explained 49.5% and 24.6% of the variance, and separation was driven primarily by storage duration (Figure 2C,D). The 9‐month fungal communities formed a distinct and compact cluster, separated from all earlier timepoints (*p*_adj_ = 0.003; Table S2). Six‐month communities differed significantly from harvest (*p*_adj_ = 0.003) and 9 months (*p*_adj_ = 0.003) but not from 3‐month communities after correction (*p*_adj_ = 0.050), with harvest and 3‐month samples remaining compositionally similar (*p*_adj_ = 0.126; Table S2). Separation by grain nitrogen content was limited relative to storage duration, with substantial overlap between nitrogen groups across ordination space (Figure 2C,D).

### 3.3 Taxonomic Composition and Succession in Stored Barley Microbiomes

**FIGURE 3:**
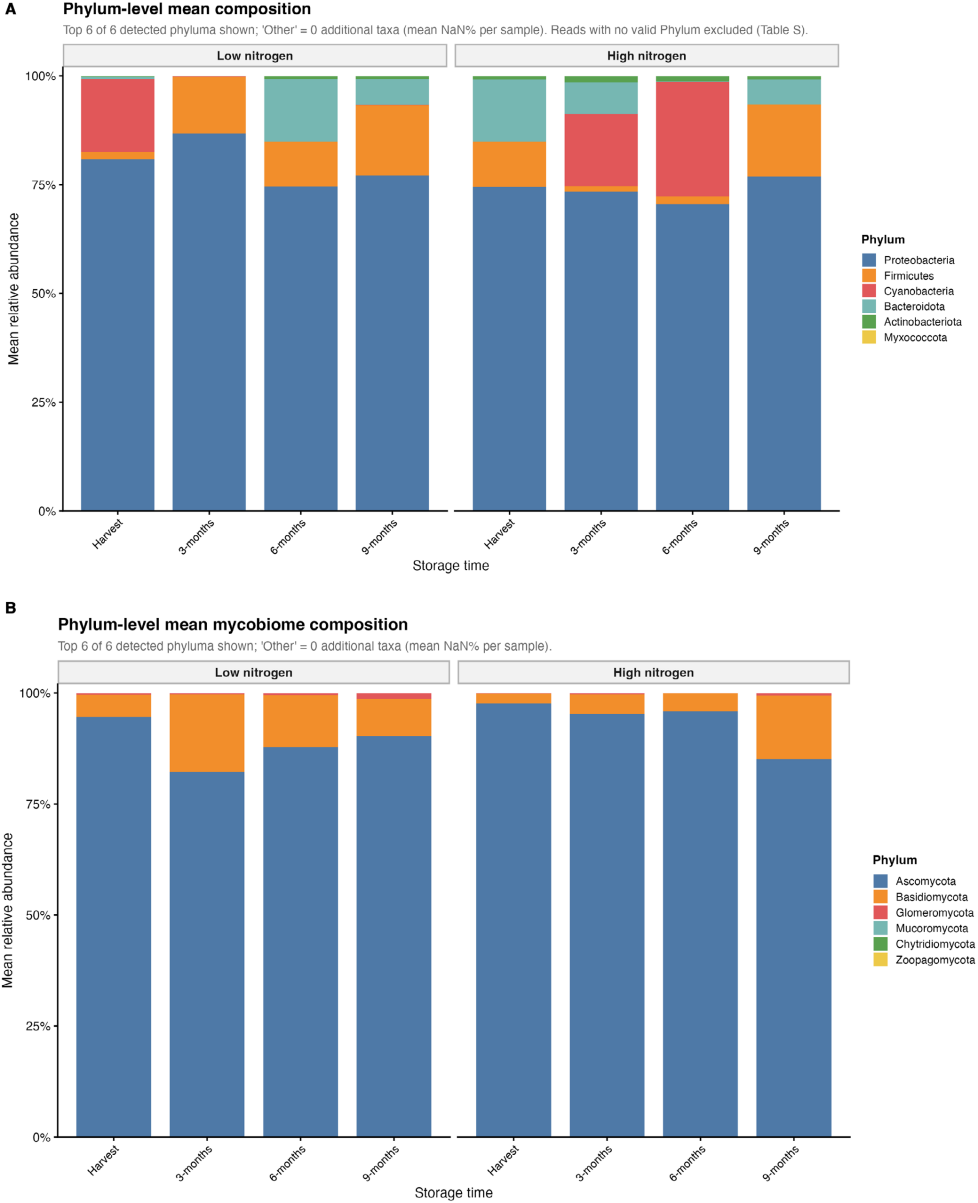
Phylum‐level mean relative abundance of the (A) bacterial and (B) fungal communities in stored barley grain across storage time and grain nitrogen content.

Bacterial communities in stored barley grain were primarily composed of a core group of phyla consistently recovered across both metataxonomic and metagenomic datasets. Analysis of the 16S rRNA amplicon data revealed six phyla across all samples. Proteobacteria represented the vast majority of the community with a mean relative abundance of 75.5% (Table S5 and Figure 3A). This was followed by Firmicutes (10.2%), Cyanobacteria (7.5%), and Bacteroidota (6.0%). Actinobacteriota and Myxococcota made only marginal contributions to the overall profile. Metagenomic sequencing was performed on a subset of eight samples, comprising one biological replicate per combination of storage duration (harvest, 3, 6, and 9 months) and grain nitrogen content (high, low). This subset corroborated the taxonomic profiles identified in the amplicon data, with Proteobacteria (91.7%), Bacteroidota (3.6%), Firmicutes (2.4%), and Actinobacteria (2.0%) identified as the dominant phyla (Figure 3A). At harvest, Firmicutes represented < 0.1% in both nitrogen groups (0.10% high‐nitrogen; 0.07% low‐nitrogen). Bacteroidota was present in both groups, comprising 7.0% in the high‐nitrogen sample and 9.1% in the low‐nitrogen sample (Table S5). Following 6 months of storage, Firmicutes relative abundance increased to 5.8% in low nitrogen samples, whereas Bacteroidetes shifted to near‐zero in high nitrogen samples. After 9 months of storage, Firmicutes accounted for 10.0% in high nitrogen samples, with Bacteroidetes exhibiting a partial recovery across both nitrogen treatments (Figure 3A). The core fungal community comprised four phyla, with Ascomycota (91.1%) and Basidiomycota (8.4%) accounting for nearly all sequences. Glomeromycota (< 0.5%) and Mucoromycota (< 0.01%) were present only at negligible proportions (Figure 3B).

### 3.4 Genus Composition and Multi‐Omics Successional Patterns

**FIGURE 4:**
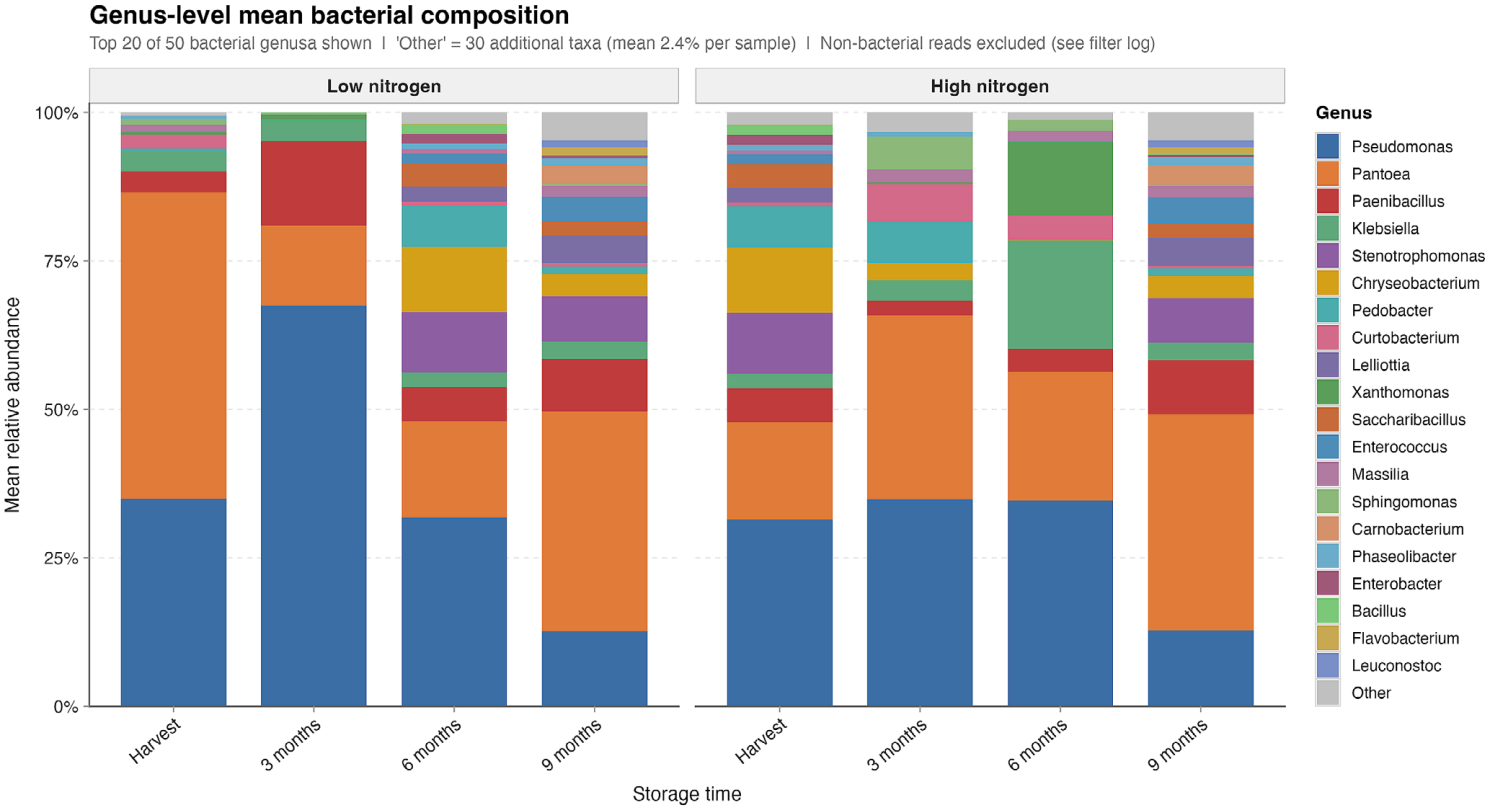
Genus‐level mean relative abundance of the bacterial community in stored barley grain across storage time and grain nitrogen content (16S rRNA gene amplicon sequencing).

The integration of metataxonomic, metagenomic and metaproteomic datasets identified 507 unique genera. Metagenomic sequencing recovered the broadest diversity with 466 bacterial genera, while 16S rRNA and ITS1 amplicons identified 71 bacterial and 128 fungal genera, respectively (Table S4). A core group of seven bacterial genera, including *Pseudomonas*, *Pantoea*, *Chryseobacterium*, *Pedobacter*, *Stenotrophomonas*, *Paenibacillus* and *Massilia*, was consistently detected across all three omics datasets (Table S4). Within the 16S rRNA dataset, *Pseudomonas* (31.1%) and *Pantoea* (28.6%) were the most prevalent members, defining the primary taxonomic structure of the barley microbiome (Figure 4).

At harvest, nitrogen availability influenced this bacterial structure. High nitrogen content grain was associated with a community characterized by *Pseudomonas* (31.6%), *Pantoea* (16.3%), *Chryseobacterium* (10.9%) and *Stenotrophomonas* (10.2%) (Figure 4). In contrast, communities in low nitrogen content barley were restricted primarily to *Pantoea* (51.0%) and *Pseudomonas* (35.0%), with other taxa largely undetected. As storage progressed, these distinct nitrogen‐associated profiles converged. At 9 months of storage, *Pantoea* relative abundance reached approximately 37% in both categories, overriding the initial differences observed at harvest (Figure 4).

**FIGURE 5:**
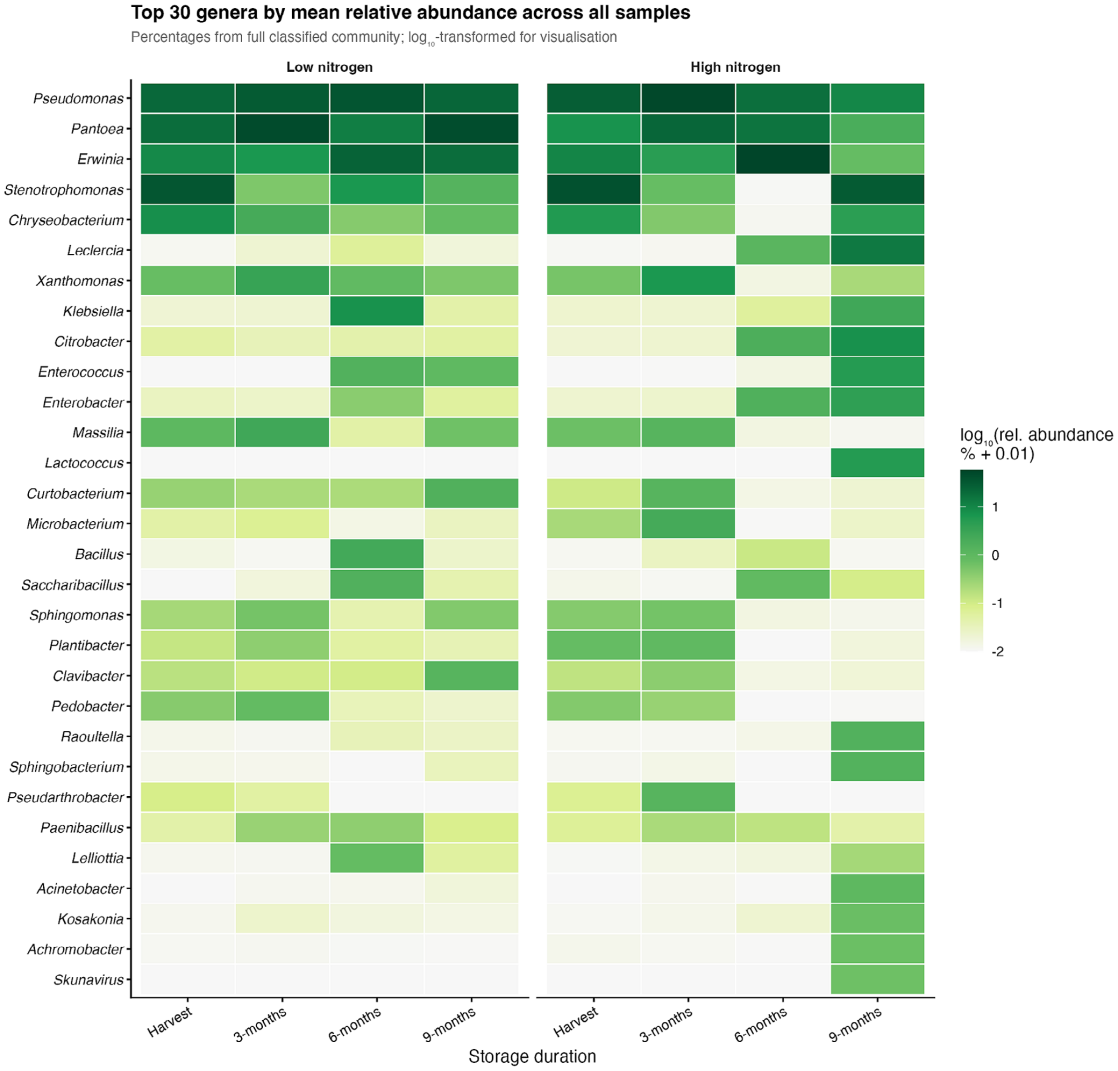
Heatmap of log_10_‐transformed relative abundance (% of classified reads) for the 30 most abundant bacterial genera identified by metagenomic sequencing (Kraken2/Bracken), shown across storage time and grain nitrogen content. Rows are ordered by mean relative abundance; color scale reflects log_10_ (% relative abundance).

**FIGURE 6:**
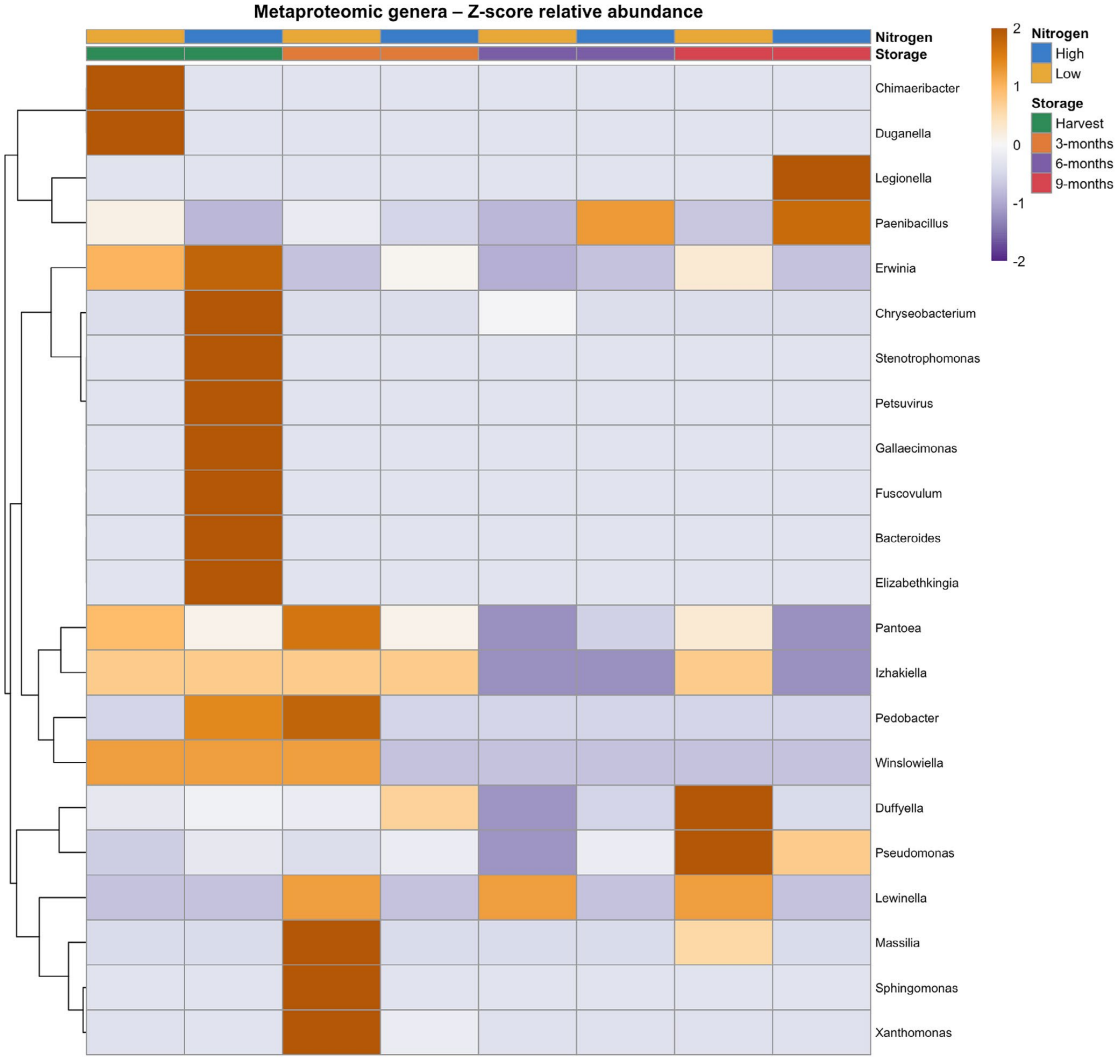
Heatmap of *Z*‐score normalized relative abundance of bacterial genera identified by metaproteomic analysis (Unipept peptide assignment) across storage time and grain nitrogen content. Rows are ordered by hierarchical clustering. Annotation bars indicate grain nitrogen content (top) and storage time (bottom).

Metagenomic analysis further resolved these taxonomic shifts by distinguishing between core and non‐core microbiome components. Of the 381 bacterial genera identified through metagenomics, 49 were classified as core members based on their presence in all samples, while the remainder were defined as non‐core (Table S4). This core was characterized by a subset including *Pseudomonas*, *Pantoea*, and *Erwinia*, which together accounted for 70%–95% of classified reads per metagenome. Within the core, *Erwinia* showed the largest fluctuation, peaking at 58.2% in the 3‐month high nitrogen sample before declining to 4.9%–25.7% at 6 months and 0.8%–6.7% at 9 months. Non‐core genera showed more variable detection patterns across the eight samples. *Saccharibacillus* and *Plantibacter* were detected in both nitrogen groups but at higher abundance in individual high nitrogen samples. *Lactococcus* (5.58%) and *Sphingobacterium* (1.45%) were detected at appreciable abundance only in the 9‐month low nitrogen sample, with trace or no detection elsewhere (Figure 5). Given the single biological replicate per storage × nitrogen combination, these patterns describe the metagenomic subset rather than supporting generalisable nitrogen‐specific or storage‐specific effects. Metaproteomic analysis corroborated these shifts; *Pseudomonas* peptides increased to approximately 70% of assigned spectra at 9 months of storage across both nitrogen categories, confirming that the taxonomic convergence observed in the DNA data is mirrored in the detectable protein pool (Table S9 and Figure 6).

Fungal ITS sequencing revealed a low‐diversity community throughout storage, while fungal taxa were undetected in metaproteomic analysis and accounted for less than 0.1% of classified reads in metagenomics. *Alternaria* was the predominant fungal genus across the study (mean 90.7%), accounting for 97.4% of the community in high grain nitrogen samples and 94.1% in low grain nitrogen samples at harvest (Figure S1). The remainder of the fungal community comprised three Basidiomycota genera: *Sporobolomyces* (mean 3.2%), *Cryptococcus* (mean 2.3%) and *Filobasidium* (mean 1.8%). At 3 months, *Sporobolomyces* was substantially more abundant in low grain nitrogen samples (10.1%) than in high grain nitrogen samples (2.0%). *Cryptococcus* and *Filobasidium* increased in relative abundance over the storage period across both grain nitrogen content groups, reaching peak abundances at 9 months. *Cryptococcus* reached 7.4% in high grain nitrogen samples and 2.7% in low grain nitrogen samples, while *Filobasidium* accounted for 3.6% and 3.8%, respectively (Figure S1).

### 3.5 Metagenome Assembly and Functional Annotation of the Barley Grain Microbiome

Assembly of the eight metagenomes yielded 274,778 contigs in total, with individual assembly sizes ranging from 78 to 180 Mb. A total of 952,951 protein‐coding sequences (CDS) were predicted across the eight assemblies, of which 706,953 were functionally annotated with eggNOG‐mapper and assigned to 23 COG categories (Table S11). The most represented category was ‘function unknown’ (S), comprising approximately 21% (167,146 entries) of all COG‐assigned proteins. Among categories with defined functions, ‘transcription’ (K; 67,581 entries) and ‘amino acid transport and metabolism’ (E; 60,147 entries) were the most abundant, followed by ‘inorganic ion transport and metabolism’ (P; 53,096 entries) and ‘carbohydrate transport and metabolism’ (G; 52,288 entries).

To compare functional profiles across sample groups, the relative abundance of CDS within each COG category was *z*‐score‐normalized and the results were visualized as a heatmap (Figure S2). Hierarchical clustering separated early‐storage samples (post‐harvest and 3 months) from late‐storage samples (6 and 9 months) along a gradient observable across multiple categories, including ‘carbohydrate transport and metabolism’ (G), ‘cell wall/membrane/envelope biogenesis’ (M), and ‘energy production and conversion’ (C) (Table S11). PERMANOVA of Bray–Curtis dissimilarities did not reveal significant differentiation of COG profiles by storage duration (*R* ^2^ = 0.599, *F*_3,4_ = 1.99, *p* = 0.256) or grain nitrogen content (*R* ^2^ = 0.031, *F*_1,6_ = 0.19, *p* = 0.675; Table S6). ANOSIM similarly returned non‐significant results for both variables (storage: *R* = 0.042, *p* = 0.424; nitrogen content: *R* = 0.000, *p* = 0.485; Table S6). No individual COG category differed significantly between storage timepoints or grain nitrogen content groups after Benjamini–Hochberg correction in Kruskal–Wallis tests (all *p*_adj_ > 0.32 for storage; all *p*_adj_ ≥ 0.50 for grain nitrogen content; Table S7). The overall COG‐level functional composition of the barley grain microbiome was therefore stable across the 9‐month silo storage period examined.

### 3.6 CAZyme Profiles

A total of 10,286 CAZymes were identified across the eight annotated metagenomes, spanning six enzyme classes and 90 families (Table S10). Glycosyltransferases (GTs) were the most encoded class, accounting for 46.4% of all annotated CAZyme sequences, followed closely by glycoside hydrolases (GHs; 44.4%), carbohydrate‐binding modules (CBMs; 5.4%), carbohydrate esterases (CEs; 2.6%), polysaccharide lyases (PLs; 0.7%), and auxiliary activities (AAs; 0.5%) collectively accounted for less than 10% of the total (Figure S3). Within the GTs, the GT2 family was the most highly encoded (1580 sequences), followed by GT4 (1130 sequences). Within the GHs, GH13 was the most represented family (1246 sequences), consistent with the starch‐degrading and glycan‐modifying capacity expected from the gram‐negative bacteria that characterize this microbiome (Table S10).

Profiling the top 20 CAZyme families after *z*‐score normalization and hierarchical clustering revealed a temporal pattern (Figure S3) broadly parallel to that observed in the COG data. The three most highly encoded families (GT2, GH13 and GT4) maintained consistent relative proportions across all storage timepoints and both grain nitrogen content groups. A separate cluster, comprising GT1, GH18 and GH23, showed elevated z‐scores at the 6‐month storage timepoint in low nitrogen content samples, where GT8 and GT1 (polysaccharide synthesis), GH18 (chitinases) and GH23 (peptidoglycan lyases/lysozymes) were jointly over‐represented (Figure S3). After 9 months of silo storage, samples from both the high and low nitrogen content groups displayed broadly negative *z*‐scores across the major CAZyme families, reflecting a reduction in the encoded carbohydrate‐active enzymatic diversity detected at that timepoint. GH3 and GH5, which encode cellulose‐ and hemicellulose‐active functions, declined between post‐harvest and 6 months before partially recovering at 9 months (Figure S3). PERMANOVA returned non‐significant results for both storage duration (*R* ^2^ = 0.656, *F*_3,4_ = 2.55, *p* = 0.105) and grain nitrogen content (*R* ^2^ = 0.049, *F*_1,6_ = 0.31, *p* = 0.737), and no individual CAZyme family reached significance after correction (all *p*_adj_ > 0.41 for storage; all *p*_adj_ > 0.84 for grain nitrogen content; Table S7).

### 3.7 Functional Analysis of Metaproteomic Data

A total of 2694 InterPro‐annotated protein hits were identified across the eight samples, mapping to 371 distinct protein families (Tables S8 and S12). The most prevalent families were outer membrane proteins (OMPs; 16.5%), followed by catalases (10.1%), porins (7.3%) and TonB‐dependent receptor‐like proteins (7.1%). Pyridine nucleotide‐disulphide oxidoreductases (3.7%), solute‐binding proteins (3.4%), flagellins (2.3%) and FAD/NAD(P)‐binding domain proteins (2.3%) made up smaller but consistently detected fractions (Figure S4). This composition is consistent with a community dominated by Gram‐negative bacteria reliant on outer membrane machinery for structural integrity and high‐affinity nutrient acquisition (Silhavy et al. 2010).

Expressed protein profiles varied with both storage duration and grain nitrogen content (Figure S4). At post‐harvest, OmpA‐like relative abundance differed between grain nitrogen content groups: high nitrogen content samples contained 26.3% OMPs compared with 16.2% in low nitrogen content samples, while catalase showed the inverse pattern (6.5% vs. 15.7%, respectively). After 6 months of silo storage, catalase representation had risen to 26.1% in high nitrogen content samples, the highest value recorded across any protein family at any timepoint, compared with 14.1% in low nitrogen content samples at the same point (Figure S4). After 3 months of storage, TonB‐dependent receptor‐like proteins accounted for 24.8% of annotated hits in low nitrogen content samples, compared with 7.2% in high nitrogen content samples at the same timepoint. Solute‐binding proteins were elevated at 6 months of storage across both nitrogen content groups (11% and 11.5% in high and low nitrogen content samples, respectively) before declining sharply at 9 months (Figure S4).

The most pronounced shift in the expressed metaproteome occurred between 6 and 9 months of silo storage. In high grain nitrogen content samples, total InterPro‐annotated protein representation fell from approximately 85% of detected peptides at 6 months of storage to approximately 25% at 9 months of storage, accompanied by a sharp decline in OMP representation from 22.9% to 4.8%. Low nitrogen content grain underwent a less severe reduction, retaining approximately 47% annotated coverage at 9 months. Despite these patterns, no individual InterPro family reached statistical significance after correction (all *p*_adj_ > 0.47 for storage; all *p*_adj_ > 0.42 for grain nitrogen content; Table S7). PERMANOVA of Bray–Curtis dissimilarities derived from metaproteomic peptide profiles (Unipept) yielded the sole significant multivariate result across all functional annotation layers: storage duration explained 53.1% of variance in expressed protein community composition (*R* ^2^ = 0.531, *F*_3,4_ = 1.51, *p* = 0.033), with no detectable effect of grain nitrogen content (*R* ^2^ = 0.153, *p* = 0.337; Table S6).

## 4 Discussion

Commercial silo storage of brewing barley for 3–9 months before malting is standard industry practice, yet the associated microbial communities have been characterized almost exclusively by culture‐based or single‐marker methods (Felšöciová et al. 2021; Bamforth 2017). The present study integrates 16S rRNA amplicon sequencing, ITS1 amplicon sequencing, metagenomics and metaproteomics across four storage timepoints and two nitrogen content groups. Storage duration was the primary driver of microbial community structure in both bacteria and fungi. Grain nitrogen content, when considered in isolation, had no detectable effect on bacterial (beta‐diversity PERMANOVA *R* ^2^ = 1.24%, *p* = 0.989) or fungal community structure (*R* ^2^ = 11.34%, *p* = 0.054). A significant interaction with storage duration was observed for bacterial communities (*R* ^2^ = 26.48%, *p* = 0.003), suggesting that nitrogen availability shapes how bacterial communities respond to prolonged storage rather than acting independently.

The fungal community appears to follow a temporal succession driven by progressive shifts in storage conditions rather than by grain nitrogen content. The dominant Basidiomycota genera *Cryptococcus* and *Filobasidium* increased in relative abundance towards 9 months across both grain nitrogen content groups. The absence of a detectable nitrogen effect on fungal structure is consistent with broader evidence that bacterial communities are more responsive than fungal communities to nitrogen and phosphorus variation in soils (Chen, Yin, et al. 2022; Li et al. 2019). The bacterial response identified here was, in contrast, interaction‐driven: grain nitrogen content shaped composition only in conjunction with storage duration, a pattern that earlier longitudinal studies of stored grain have not resolved because the substrate was treated as compositionally uniform (Solanki et al. 2019).

Core microbial communities in stored grain are increasingly recognized as relevant to seed health and resistance to spoilage (Solanki et al. 2021). In this study, a seven‐genus bacterial core comprising *Pseudomonas*, *Pantoea*, *Chryseobacterium*, *Pedobacter*, *Stenotrophomonas*, *Paenibacillus*, and *Massilia* was consistently detected across the amplicon, metagenomic, and metaproteomic datasets. The congruence of these taxa across all three omics layers is consistent with persistent residency rather than transient contamination and aligns with bacterial genera frequently reported in field‐grown barley and the broader cereal seed microbiome (Bianco et al. 2018; Felšöciová et al. 2021; Bziuk et al. 2021; Simonin et al. 2022). Although likely inherited from the parent plant and maintained throughout storage, the relative abundance of core members shifted over time and between grain nitrogen content groups.

At harvest, differences between nitrogen treatments were driven less by the abundance of *Pseudomonas*, which was similar in high nitrogen (31.6%) and low nitrogen (35.0%) grain, and more by shifts in community structure and the prominence of *Pantoea*. High‐nitrogen grain supported a more heterogeneous assembly, with *Chryseobacterium* (10.9%) and *Stenotrophomonas* (10.2%) occurring alongside *Pantoea* (16.3%) and *Pseudomonas* (31.6%). In contrast, low nitrogen grain was dominated by *Pantoea* (51.0%) and *Pseudomonas* (35.0%), with few additional taxa detected (Figure 4). The most pronounced compositional change occurred between the 6‐and 9‐month timepoints, with metaproteomic data at these timepoints detecting outer membrane proteins, catalases and porins consistent with continued activity of the Gram‐negative core. This trajectory is consistent with a transition from resource‐rich to oligotrophic conditions, in which earlier colonizers are partially displaced by stress‐tolerant, endospore‐forming genera such as *Paenibacillus* (Solanki et al. 2021). Metagenome‐assembled genome analysis of the same samples supports this trend, with declines in *Pseudomonas* and *Chryseobacterium* recovery towards later timepoints (Tshisekedi et al. 2024). Tracking this transition towards stress‐tolerant taxa may offer a practical indicator for monitoring storage stability and refining post‐harvest management.

Beyond the persistent core, non‐core taxa contributed variable, sample‐specific signals to the metagenomic profiles. Their detection patterns were not consistent across the eight samples, and several genera were observed at appreciable abundance in only one or two samples, limiting any generalisable nitrogen‐by‐storage interpretation. Within these limits, a few taxa with plausible functional relevance to grain storage warrant description. *Plantibacter*, a genus typically associated with plant tissues, was detected in both nitrogen groups but was more prominent in high grain nitrogen samples (up to 0.94% at 6 months). *Saccharibacillus*, which carries known polysaccharide‐degrading capacity, showed similar variability: it was detected in both nitrogen groups but showed its highest relative proportion at 6 months in high grain nitrogen samples (1.57%), compared to a markedly lower proportion in low grain nitrogen samples (≤ 0.08%). *Pedobacter*, a member of the persistent core, was detected across both nitrogen groups throughout storage. The clearest single‐sample signals were observed at 9 months in low grain nitrogen samples, where *Lactococcus* (5.58%) and *Sphingobacterium* (1.45%) represented a higher relative proportion at this timepoint but were not detected at comparable levels elsewhere. The detection of lactic acid bacteria in late‐stage stored cereals is consistent with shifts towards anaerobic, nutrient‐limited microenvironments documented in moist barley and silage systems (Duniere et al. 2017; Borling Welin et al. 2015). Because grain nitrogen content was confounded with storage time within the metagenomic subset, these patterns describe the eight samples rather than support a generalisable nitrogen‐driven succession. Whether endogenous grain composition shapes non‐core recruitment in stored barley is a tractable question, but it requires a balanced metagenomic design with replication across each storage × nitrogen combination to test directly.

The fungal community was overwhelmingly dominated by a single genus, *Alternaria*, which consistently accounted for over 90% of fungal ITS reads across all storage times and nitrogen content groups. The prevalence of this genus has been previously observed in stored barley and is of concern, as *Alternaria* species are known producers of numerous mycotoxins that pose a risk to food and feed safety (Felšöciová et al. 2021; Escrivá et al. 2017). The low diversity and absence of other common field fungi, such as *Fusarium*, *Penicillium*, and *Aspergillus*, may reflect the superior competitive ability of *Alternaria* under typical grain storage conditions, which ideally involve temperatures between 15°C and 20°C and moisture content below 15% (Brito et al. 2022). The limited detection of fungal taxa in the metagenomic and metaproteomic datasets likely reflects technical limitations (Jiao et al. 2021). This underrepresentation of the mycobiome often arises from the high bacterial‐to‐fungal biomass ratio in cereal grains, alongside the inherent difficulty of extracting and sequencing large eukaryotic genomes and proteins from complex environmental samples (Jo et al. 2020; Setubal 2021).

Functional annotation of the assembled metagenomes identified a diverse range of functional categories. Genes related to transport, metabolism, and transcription were among the most abundant in the dataset. Although taxonomic successions were observed, multivariate testing indicated that the functional profile of the microbiome remained stable. Neither storage duration nor grain nitrogen content significantly influenced the COG or CAZyme compositions (COG PERMANOVA storage *p* = 0.256, nitrogen *p* = 0.675; CAZyme PERMANOVA storage *p* = 0.105, nitrogen *p* = 0.737).

The prevalence of GH13 enzymes is consistent with a community of starch‐utilizing bacteria feeding on the barley endosperm, while the high frequency of GT2 and GT4 families reflects the structural requirements of the Gram‐negative bacteria that provide most of the biomass (Drula et al. 2022). In grain with a low nitrogen content, the detection of GH18 and GH23 sequences at 6 months storage suggests possible antagonistic potential (Veliz et al. 2017). These CAZyme families are associated with the degradation of fungal and bacterial cell walls; their presence hints that the community may be genetically equipped for inter‐microbial competition (Chen, Liang, et al. 2022). Such a shift implies that under low‐nitrogen conditions, specific taxa might possess the metabolic potential to protect their ecological niche from competitors or spoilage organisms. Additionally, changes in the copy number of GH3 and GH5 sequences were observed. These enzymes are known to break down cellulose and complex plant fibers (Lombard et al. 2014). This points towards a possible shift in the community towards the breakdown of more recalcitrant substrates during the late storage phase (Li et al. 2025).

Metaproteomic profiling provides a resolution of microbial activity that DNA‐based datasets cannot capture, identifying protein families associated with structural integrity and environmental sensing. In this study, OMPs, porins and TonB‐dependent receptors were detected. These systems are known to facilitate specialized nutrient uptake and sensing in Gram‐negative bacteria (Achouak et al. 2001; Nikaido 2003). This points towards a potential community reliance on these systems to navigate the storage environment. Analysis revealed that these expressed protein profiles changed significantly over time, with storage duration explaining over half of the observed variance (*p* = 0.033), whereas grain nitrogen content had no discernible effect. While the genetic potential of the microbiome remains stable, the expressed proteins suggest a physiological response that tracks environmental shifts during storage (Li et al. 2023). Specifically, the higher frequency of TonB‐dependent receptors observed in low nitrogen content grain at 3 months indicates an early transition towards nutrient scavenging. Since these systems are known to facilitate the uptake of iron and carbohydrates in substrate‐poor conditions, their presence suggests the community is switching from surface colonization towards stress defense and resource acquisition (Noinaj et al. 2010; Nikaido 2003). Furthermore, an increase in catalase detection was observed at 6 months storage. This enzyme is known to mitigate oxidative stress caused by desiccation or fungal metabolites like alternariol (Crudo et al. 2021). Such an increase is consistent with a physiological response to the storage environment. By 9 months, the abundance of proteins declined, implying a potential shift into a dormant or viable‐but‐non‐culturable state as storage stress persists (Shade et al. 2017). Together, these results reflect a transition from active colonization to a widespread reduction in metabolic activity during prolonged storage.

## 5 Conclusion

The results of this study indicate that storage duration was the dominant driver of microbial community structure across all datasets, with grain nitrogen content exerting a detectable effect only through its interaction with storage time in bacterial communities. Bacterial communities appear to respond to the interaction of storage time and grain nitrogen content, whereas fungal communities follow storage duration alone. A functional distinction between omic datasets is evident, where the encoded functional potential remains relatively stable, while the expressed metaproteome tracks storage duration and exhibits a physiological progression from surface colonization at harvest to nutrient scavenging and a reduction in metabolic activity by the final timepoint of 9 months. The consistent presence of mycotoxigenic *Alternaria* across all samples represents a food safety concern that highlights the need for optimized storage strategies, while the lower frequency of *Fusarium*, *Aspergillus*, and *Penicillium* may reflect the competitive structure of this specific storage system. Acknowledging certain limitations, the more detailed information for bacteria likely reflects the higher bacterial‐to‐fungal biomass ratio and known challenges in detecting fungal results from shotgun datasets. Since DNA‐based methods cannot distinguish between various physiological states, interpretation was supported by metaproteomic results where possible. While a modest sample size for metagenomic and metaproteomic components may limit the power of specific family level analyses, the observed trends suggest that multi‐omic designs are necessary to capture the functional response of stored grain microbiomes, which single omic studies may under‐report.

## Author Contributions

**Tim Van Den Bossche:** methodology, data curation, writing – review and editing. **Kalonji A. Tshisekedi:** writing – original draft, writing – review and editing, data curation. **Pieter De Maayer:** supervision, writing – review and editing, methodology. **Angela Botes:** conceptualization, writing – review and editing, supervision, project administration, funding acquisition. **Lennart Martens:** software, methodology.

## Funding

This project was funded by the National Research Foundation (NRF) and Anheuser‐Busch InBev SA/NV (AB‐InBev).

## Conflicts of Interest

The authors declare no conflicts of interest.

## Supporting information


**Figure S1:** Genus‐level mean relative abundance of the fungal community in stored barley grain across storage time and grain nitrogen content (ITS1 amplicon sequencing, option B; *n* = 23). Top 20 of 128 detected genera are shown; remaining taxa are pooled as ‘Other’. Colors indicate genus.
**Figure S2:** Heatmap of *Z*‐score normalized relative abundance of Clusters of Orthologous Groups (COG) functional categories identified by shotgun metagenomic sequencing (*n* = 8). Rows are ordered by hierarchical clustering; columns are ordered by storage time and grain nitrogen content.
**Figure S3:** Heatmap of *Z*‐score normalized relative abundance of the top 20 Carbohydrate‐Active enZyme (CAZyme) families identified by shotgun metagenomic sequencing (*n* = 8). CBM, carbohydrate‐binding modules; CE, carbohydrate esterases; GH, glycoside hydrolases; GT, glycosyltransferases. Rows are ordered by hierarchical clustering; columns are ordered by storage time and grain nitrogen content.
**Figure S4:** Mean relative abundance of InterPro protein families identified by metaproteomic analysis of stored barley grain (% of all annotated spectral hits; *n* = 8). Top 10 families are shown; remaining families are pooled as ‘Other’. Results are shown for high‐nitrogen and low‐nitrogen grain across four storage time points.

**Table S1:** Alpha diversity—bacterial and fungal communities (rarefaction option B). Alpha diversity indices for bacterial (16S rRNA amplicon sequencing, rarefaction option B) and fungal (ITS1 amplicon sequencing, option B) communities in stored barley grain. Observed: number of observed ASVs/OTUs; Chao1: estimated richness; SE Chao1: standard error of the Chao1 estimate; Shannon: Shannon entropy. Nitrogen: grain nitrogen content (High/Low).
**Table S2:** Beta diversity statistics and PCoA coordinates—bacterial and fungal communities. Beta diversity analyses based on Bray–Curtis dissimilarity for bacterial (16S rRNA gene amplicon sequencing, option B; *n* = 22) and fungal (ITS1 amplicon sequencing, option B; *n* = 23) communities. PERMANOVA: adonis2, 999 permutations; *R* ^2^: proportion of variance explained; *F*: pseudo‐*F* statistic. ANOSIM: *R* statistic (0 = no separation, 1 = complete separation). Betadisper: ANOVA test for homogeneity of multivariate dispersion. Pairwise PERMANOVA *p*‐values corrected using the Benjamini‐Hochberg (BH) method. Significance: ***p < 0.001, **p < 0.01, *p < 0.05, ns = not significant.
**Table S3:** Dunn post hoc pairwise comparisons—fungal alpha diversity (ITS1). Kruskal–Wallis test results and Dunn post hoc pairwise comparisons of fungal alpha diversity indices (ITS1 amplicon sequencing, option B; *n* = 23) across storage time groups. Kruskal–Wallis: Chi^2^: test statistic; df: degrees of freedom; *p*: *p*‐value. Dunn post hoc: *Z*: standardized test statistic; *p*_adj (BH): Benjamini‐Hochberg‐adjusted *p*‐value. Significance: ***p < 0.001, **p < 0.01, *p < 0.05, ns = not significant. Bold: *p*_adj < 0.05.
**Table S4:** Core and non‐core bacterial genera identified by metagenomic sequencing (Kraken2/Bracken). Core and non‐core bacterial genera identified by metagenomic sequencing (Kraken2/Bracken). Core genera are present in all eight metagenome samples; non‐core genera are absent in at least one sample. Columns represent individual samples coded by storage time and nitrogen content. Values are raw read counts.
**Table S5:** Metagenome assembly and structural annotation metrics.
**Table S6:** PERMANOVA and ANOSIM—functional and metaproteomic datasets. PERMANOVA (adonis2) and ANOSIM results testing the effect of storage time and grain nitrogen content on functional community profiles (COG, CAZyme, InterPro) and metaproteomic taxonomic composition (Unipept). Bray–Curtis dissimilarity, 999 permutations. *R* ^2^: variance explained; *F*: pseudo‐*F* statistic; *R*: ANOSIM statistic (0 = no separation, 1 = complete separation). Significant: *p* < 0.05. Bold: *p* < 0.05.
**Table S7:** Kruskal–Wallis tests—per‐feature relative abundance. Kruskal–Wallis tests on per‐feature relative abundances for metagenomic functional datasets (COG, CAZyme, InterPro) and metaproteomic taxonomic data (Unipept), testing for effects of storage time and grain nitrogen content. *p*_adj (BH): Benjamini‐Hochberg‐adjusted *p*‐value. Significant_adj: *p*_adj < 0.05. Bold rows: significant after BH correction.
**Table S8:** InterPro protein family distribution—metaproteomic analysis. Relative abundance of InterPro protein families from metaproteomic analysis of stored barley grain. RelAbund_pct: % of all InterPro‐annotated spectral hits per sample assigned to each protein family (sums to 100% per sample). A single protein may contribute to multiple families. Sample codes: H, Harvest; HN, high nitrogen; LN, low nitrogen; T1, 3‐months; T2, 6‐months; T3, 9‐months.
**Table S9:** Metaproteomic taxonomic assignment—genus level (Unipept). Relative abundance of microbial genera identified by Unipept taxonomic analysis of metaproteomic data from stored barley grain. RelAbund_pct: % of all taxonomically assigned peptides per sample attributed to each genus (sums to 100% per sample; one peptide maps to one genus). *n* = 1 per condition; SD not applicable. Sample codes: H, Harvest; HN, high nitrogen; LN, low nitrogen; T1, 3‐months; T2, 6‐months; T3, 9‐months.
**Table S10:** CAZyme family distribution—metagenomic sequencing. Relative abundance of Carbohydrate‐Active enZyme (CAZyme) families from shotgun metagenomic sequencing of barley grain. RelAbund_pct: % of all CAZyme‐annotated sequences per sample (sums to 100% per sample). Enzyme classes: AA, auxiliary activities; CBM, carbohydrate‐binding modules; CE, carbohydrate esterases; GH, glycoside hydrolases; GT, glycosyltransferases; PL, polysaccharide lyases. Sample codes: H, Harvest; HN, high nitrogen; LN, low nitrogen; T1, 3‐months; T2, 6‐months; T3, 9‐months.
**Table S11:** COG functional category distribution—metagenomic sequencing. Relative abundance of Clusters of Orthologous Groups (COG) functional categories from shotgun metagenomic sequencing of barley grain. RelAbund_pct: % of all annotated CDS per sample assigned to each category (sums to 100% per sample). Group means computed across replicates per storage time × nitrogen condition. Sample codes: H, Harvest; HN, high nitrogen; LN, low nitrogen; T1, 3‐months; T2, 6‐months; T3, 9‐months.
**Table S12:** InterPro protein family relative abundance—mean per storage time and nitrogen condition. Mean relative abundance (% of all InterPro‐annotated spectral hits) of the 20 most abundant InterPro protein families from metaproteomic analysis of stored barley grain, grouped by storage duration and grain nitrogen content. Values are single‐replicate means (*n* = 1 per condition). Families are ranked by overall mean abundance. Outer membrane protein (OMP) at 9 months in the high‐nitrogen condition represents 4.81% of annotated hits; this value is not visually resolved in Figure S4.

## Data Availability

The raw reads used to reconstruct the MAGs have been deposited to the NCBI Sequence Read Archive under accession SRP479463. The 16S rRNA gene and ITS amplicon sequencing reads have been deposited to the NCBI Sequence Read Archive under BioProject accession PRJNA1029034. The mass spectrometry proteomics data have been deposited to the ProteomeXchange Consortium (http://proteomecentral.proteomexchange.org) via the PRIDE partner repository (Perez‐Riverol et al. 2025) with the dataset identifier PXD046266. In this repository, the metadata has also been deposited in SDRF‐Proteomics format (Dai et al. 2021), generated with the lesSDRF tool (Claeys et al. 2023).
