# Supplementary material for: Temporal and Functional Profiling of the Microbiome of High and Low Nitrogen Content Barley Seed in Silo Storage
Source: Food Sci Nutr. 2026 Aug 10;14(8):e72179. doi: 10.1002/fsn3.72179 (PMC13458027; doi:10.1002/fsn3.72179)
Supplement: Supplementary file 1 [file FSN3-14-e72179-s001.zip › captions-supp-figures-Tshisekedi-et-al.docx]

Figure S1. Genus-level mean relative abundance of the fungal community in stored barley grain across storage time and grain nitrogen content (ITS1 amplicon sequencing, option B; n = 23). Top 20 of 128 detected genera are shown; remaining taxa are pooled as 'Other'. Colours indicate genus.

Figure S2. Heatmap of Z-score normalised relative abundance of Clusters of Orthologous Groups (COG) functional categories identified by shotgun metagenomic sequencing (n = 8). Rows are ordered by hierarchical clustering; columns are ordered by storage time and grain nitrogen content.

Figure S3. Heatmap of Z-score normalised relative abundance of the top 20 Carbohydrate-Active enZyme (CAZyme) families identified by shotgun metagenomic sequencing (n = 8). GH: glycoside hydrolases; GT: glycosyltransferases; CBM: carbohydrate-binding modules; CE: carbohydrate esterases. Rows are ordered by hierarchical clustering; columns are ordered by storage time and grain nitrogen content.

Figure S4. Mean relative abundance of InterPro protein families identified by metaproteomic analysis of stored barley grain (% of all annotated spectral hits; n = 8). Top 10 families are shown; remaining families are pooled as 'Other'. Results are shown for high-nitrogen and low-nitrogen grain across four storage time points.
